# Antibiotic affects the gut microbiota composition and expression of genes related to lipid metabolism and myofiber types in skeletal muscle of piglets

**DOI:** 10.1186/s12917-020-02592-0

**Published:** 2020-10-16

**Authors:** Honglin Yan, Bing Yu, Jeroen Degroote, Thomas Spranghers, Noémie Van Noten, Maryam Majdeddin, Mario Van Poucke, Luc Peelman, Jo De Vrieze, Nico Boon, Ingrid Gielen, Stefaan De Smet, Daiwen Chen, Joris Michiels

**Affiliations:** 1 Animal Nutrition Institute, Sichuan Agricultural University, Key Laboratory of Animal Disease-Resistance Nutrition, Ministry of Education, China, Ya’an, 625014 People’s Republic of China; 2 Laboratory for Animal Nutrition and Animal Product Quality, Department of Animal Sciences and Aquatic Ecology, Ghent University, Coupure Links 653, 9000 Ghent, Belgium; 3 School of Life Science and Engineering, Southwest University of Science and Technology, Mianyang, 621010 People’s Republic of China; 4 Department of Nutrition, Genetics and Ethology, Ghent University, Heidestraat 19, 9820 Merelbeke, Belgium; 5 Center for Microbial Ecology and Technology (CMET), Ghent University, Coupure Links 653, 9000 Ghent, Belgium; 6 Department of Medical Imaging and Small Animal Orthopaedics, Ghent University, Salisburylaan 133, 9820 Merelbeke, Belgium

**Keywords:** Tylosin, Intramuscular fat content, Lipid metabolism, Gut microbiota, Skeletal muscle, Piglets

## Abstract

**Background:**

Early-life antibiotic administration is known to affect gut microbiota and host adiposity, but the effects of antibiotic exposure on skeletal muscle properties remain unknown. The present study evaluated the changes in skeletal muscle properties including myofiber characteristics and composition, as well as intramuscular fat (IMF) content in skeletal muscle of piglets when exposed to a tylosin-containing diet.

**Results:**

A total of 18 piglets (28 days of age) were randomly allocated into two groups: control basal diet (Control) and Control + 100 mg tylosin phosphate/kg of feed (Antibiotic). The trial lasted for 39 days. High-throughput amplicon sequencing revealed that no significant difference in initial gut microbiota composition was existed between Control and Antibiotic groups. Antibiotic administration increased body weight and growth rate and decreased feed to gain ratio of pigs (*P* < 0.05). The carcass lean and fat volumes of pigs were increased by the tylosin administration (*P* < 0.05). Antibiotic treatment increased myofiber density and the expression of genes related to type I and type IIb myofibers in *longissimus* muscle (*P* < 0.05). The IMF content in *longissimus* muscle was increased by antibiotic exposure (*P* < 0.05). Antibiotic administration increased expression of genes related to fatty acid uptake and de novo synthesis, and decreased expression of genes related to triglyceride hydrolysis (*P* < 0.05). Tylosin administration affected taxonomic distribution and beta diversity of the caecal and colonic microbiota of piglets.

**Conclusion:**

These results confirm that the growth performance, myofiber composition and muscle lipid metabolism are affected by antibiotic administration, which may be associated with an altered gut microbiota, suggesting that the gut microbiota could be served as a potential target for modulating skeletal muscle properties of host.

## Background

Gut microbiota have been shown to play a causal role in modulating lipid metabolism in liver and adipose tissues in mice [1–3]. Skeletal muscles are also major sites of lipid metabolism [4]. Previous studies showed that the absence of gut microbiota enhanced fatty acid catabolism in skeletal muscle and reduced skeletal muscle mass of mice [3, 5]. In addition, the composition of myofiber types in skeletal muscle was found to be regulated by gut microbiota [6]. There are four major fiber types in skeletal muscle characterized by the expression of the slow/I, IIa, IIx, and IIb myosin heavy chain (MYH) isoforms, encoded by *MYH7*, *MYH2*, *MYH1*, and *MYH4* genes, respectively [6]. Germ free mice have been shown to exhibit reduced transcription of *MYH2*, *MYH1*, and *MYH4* genes in skeletal muscle [5]. These evidences suggest that the lipid metabolism and myofiber development of skeletal muscle are associated with gut microbiota signature. Moreover, the expression pattern of lipid metabolism-related genes, which are required for de novo fatty acid synthesis [acetyl-CoA carboxylase alpha (*ACACA*) and fatty acid synthase (*FASN*)], fatty acid uptake [lipoprotein lipase (*LPL*) and CD36 molecule (*CD36*)], and lipolysis [carnitine palmitoyl-transferase 1B (*CPT1B*) and patatin-like phospholipase domain containing 2 (*PNPLA2*)], in skeletal muscle could be transferred from pig donors to mice recipients by fecal microbiota transplantation [7]. This supports the contention that gut microbiota may become a new target in the regulation of lipid metabolism and myofiber development in skeletal muscle.

Antibiotics administered at sub-therapeutic doses have been used as growth promotor and a means of microbiota manipulation in livestock for over half a century [8]. The association between antibiotic exposure and host physiology has received renewed interest, acknowledging the critical role of gut microbiota in host physiology deciphered in recent years. Antibiotics administered at both low dosage and pharmaceutical level alter the gut microbiome and host metabolism, as indicated by increased adiposity, increased insulin resistance and accelerated development of type 1 diabetes [9–11]. The shifted gut microbiota, not the antibiotics per se, were found to be responsible for metabolic changes, as demonstrated by the ability to replicate metabolic changes in germ free recipients inoculated with the antibiotic-selected bacteria [11, 12]. An early study in chickens showed that the growth promotion only appeared in conventional chickens instead of germ-free ones when exposed to in-feed antibiotics, underpinning the role of the gut microbiota [13]. Importantly, effects of antibiotic exposure to animals in early life on both growth and metabolism were greater than if the exposure happened in later life [10, 14]. In Chinese swine production, piglets are frequently exposed to antibiotics during post-weaning period as the weaning stress of piglets can be alleviated by the inclusion of antibiotics in feed [15]. Tylosin phosphate is one of the most commonly used antimicrobial growth promoters in pigs’ starter diet, and shifts in both high abundant and low abundant taxa of swine gut microbiota were observed in response to the administration of tylosin [16]. The recent murine study has shown that tylosin treatment during early life profoundly alters the metabolic phenotypes of host [17]. However, the effects of post-weaning tylosin intervention on lipid metabolism and myofiber development in piglets remain unknown.

It is our hypothesis that tylosin dietary administration may modulate the gut microbiota composition, and that shifts in the gut microbiota will elicit alterations of carcass composition and skeletal muscle properties in weaning piglets. This study was designed to explore the effect of microbiota perturbation driven by a single in-feed antibiotic on lipid metabolism and myofiber composition of skeletal muscle of piglets.

## Methods

### Animal housing, treatments and sample collection

The study was conducted in accordance with the European recommendations for the protection of animals used for agricultural research (EU Directive 91/630/EEG and 98/58/EG).

Eighteen piglets (Topics hybrid × Piétrain) weaned at 18 days of age were purchased from a local commercial farm (Ghent, Belgium) and adjusted to solid feed for 10 days, and all piglets had no access to antibiotic during the 10-day adaption period. At 28 days of age (d0 of the trial), piglets were randomly allocated to receive either a control diet (*n* = 9) or an antibiotic-containing diet (*n* = 9; tylosin phosphate, 100 mg/kg diet) by using computer-generated randomization lists. Treatments were balanced for weight, gender and litter. One investigator was responsible for randomization whilst the other investigators remained blinded to the group allocation until all measurements were completed and recorded. During the entire trial, all the piglets were housed individually and had free access to water, and were fed four equal meals daily. The daily feed allowance for each piglet was adjusted according to average body weight (BW), measured weekly, following the NRC 2012 eq. [ME intake (kcal/day) = − 783.5 + 315.9× BW -5.7685 × BW^2^]. The diet (Additional file Table S1) was formulated to meet or exceed the piglets’ requirements according to the Centraal Veevoeder Bureau (CVB, The Netherlands). At d0 of the trial, fecal samples were collected and stored at − 80 °C. After 39 days, piglets were weighed and euthanized by intra-peritoneal pentobarbital (90 mg/kg BW) injection, followed by exsanguination. Immediately post-mortem, serum samples, digesta from caecum and mid-colon, and *longissimus* muscle at 10th to 12th *thoracic vertebrae* were collected, flash-frozen in liquid nitrogen, and stored at − 80 °C. The *longissimus* muscle was collected at the 5th to 9th *thoracic vertebrae* to measure intramuscular fat (IMF) content, and a small piece (2 cm × 1 cm × 1 cm) of *longissimus* muscle at the 10th to 12th *thoracic vertebrae* was fixed in 10% formaldehyde buffer for histological examinations. The muscle samples taken out from each piglet were weighed to correct carcass composition calculations. The average daily gain, feed intake and feed to gain ratio were calculated for the period d 0-d 39.

### Carcass composition, IMF content and serum metabolite concentrations

The computed tomography scanning was adopted to measure carcass composition of piglets, and was carried out as described in Supplemental experimental procedures (Additional file S1.1). The IMF content in *longissimus* muscle was measured using Soxhlet extraction method. Briefly, after thawing at 4 °C for 24 h, adhering adipose and connective tissue were removed from the *longissimus* muscle which was then ground and analyzed for moisture and fat. Moisture was determined by drying at 105 °C to reach a constant weight. Petroleum ether fat extractions were conducted for 12 h on the resultant dried products using the Soxhlet extraction apparatus. The IMF content was determined in duplicate and expressed as percentage of wet muscle weight [18]. Serum non-esterified fatty acids (NEFA) were measure with the WAKO kit (Neuss, Germany), serum glucose was determined with the Glucose liquiUV mono kit (Human, Wiesbaden, Germany) and serum triglyceride was analyzed with a kit from Human (Wiesbaden, Germany). Serum total cholesterol, high density lipoprotein (HDL), very-low density lipoprotein (VLDL) were determined with liquicolor kits (Human, Wiesbaden, Germany). The mean intra- and inter-assay variabilities for NEFA, glucose, and total cholesterol were 2.4 and 4.8%, 2.0 and 4.1%, and 1.9 and 3.5%, respectively. Intra-assay and inter-assay CVs for HDL and VLDL were 0.9 and 1.3% and 1.1 and 1.8%, respectively. Serum low density lipoprotein (LDL) concentration was calculated by the Friedewald eq. [LDL = total cholesterol - HDL - VLDL] [19].

### Histological analysis of skeletal muscle

Skeletal muscle immersed in the formalin solution was dehydrated and embedded in paraffin wax. Three slices with 3-μm thickness of each sample were cut and stained with hematoxylin and eosin to observe the morphology of muscle. The stained slices were photographed using an Olympus CX41 microscope at 40 × magnification. The Olympus Cell B software (Olympus) was used to measure myofiber characteristics. Muscle fibers from 5 random fields of each sample were chosen to measure and calculate the fiber diameter and cross-sectional area. The total fiber number of each field was counted and was converted to fiber density by dividing the area of the field. Fiber density was expressed as total fiber number per mm^2^ muscle.

### Reverse transcription quantitative PCR (RT-qPCR)

The methods of RNA extraction, cDNA synthesis and primers picking are described in Supplemental experimental procedures (Additional file S1.2). The RT-qPCR was performed on a CFX96 Touch Real-Time PCR Detection System (Bio-Rad Laboratories, Inc.) using the KAPA SYBR® FAST qPCR Kit (Kapa Biosystems, Inc., Wilmington, MA, USA). Each reaction consisted of 5 μl 2x KAPA SYBR FAST qPCR Kit Master Mix, 0.5 μl forward primer (5 μM), 0.5 μl reverse primer (5 μM), 2 μl Milli-Q water and 2 μl cDNA template in a total volume of 10 μl. The thermal cycling conditions were as follows: an initial denaturation and enzyme activation step at 95 °C for 3 min, then forty cycles of denaturation/ annealing/extension and data acquisition (95 °C for 20 s, 40 s at annealing temperature depending on primer), and melt curve analysis from 70 °C to 90 °C with 0.5 °C increment every 5 s. Five points of 4-fold serial dilutions of cDNA were included in each run to obtain the PCR efficiency by generating a standard curve. In the current study, PCR amplification efficiencies consistently ranged from 90 to 110% and were used to convert the Cq values into raw data. The *TBP*, *TOP2B* and *ACTIN* were identified as the three most stably expressed reference genes in the *longissimus* muscle of pigs [20, 21]. Therefore, *TBP*, *TOP2B* and *ACTIN* were used as reference genes in this study to normalize the raw data of RT-qPCR. The relative expression was expressed as a ratio of the target gene to the geometric mean of the reference genes, then the highest expressed samples were used as calibrator for normalizing the raw data. All the primers used in this study are listed in Additional file Table S2.

### Genomic DNA extraction and microbiota analysis

DNA extractions from fecal pellets, caecum content and colon content were carried out as previously described [22]. The DNA quality was evaluated visually by agarose gel electrophoresis. The V3-V4 region of the 16S rRNA gene was amplified using the forward primer 341F (5′-NNNNNNNNNNTCCTACGGGNGGCWGCAG-3′) and the reverse primer 785R (5′-NNNNNNNNNNTGACTACHVGGGTATCTAAKCC-3′) for each sample [23]. The resulting products were pooled and purified with Agencourt AMPure XP beads (Beckman Coulter, Brea, CA) followed by additional purification using MinElute PCR Purification Kit (Qiagen, Venlo, The Netherlands). The Illumina libraries were constructed by purified pooled amplicon DNA with the Ovation Rapid DR Multiplex System 1–96 (NuGEN, San Carlos, CA), and were sent to LGC Genomics GmbH (Berlin, Germany) for sequencing on the Illumina Miseq platform. The Illumina data processing were carried out as described in Supplemental experimental procedures (Additional file S1.3) by the Mothur software [24]. We obtained the OTU abundance table and the OTU taxonomic assignment table from Mothur software, and the subsequent processing of these two tables was performed using R studio v3.4.1 [25]. The alpha diversity indices of communities were calculated using Community Ecology Package vegan [26]. To minimize the biases caused by sequencing depth between samples, the number of sequences per sample was randomly subsampled to the minimum sequencing depth. The Bray Curtis dissimilarity index as an indicator of beta diversity between samples was calculated using the *distance* function (phyloseq) [27]. The NMDS plots visualizing the samples based on Bray Curtis distance matrix and the heatmaps representing the relative abundance of genus were made using the corresponding functions in R studio v3.4.1.

### Statistics analysis

The sample size was calculated to be 9 animals given a power of 80% to detect a difference of 10% in growth rate, with a standard deviation of 7.5% of the means at an α value of 0.05.

Piglet was considered the experimental unit for all analyses (*n* = 9 per treatment), and all data are presented as mean ± SE. The parameters host growth traits, carcass composition, serum metabolites, skeletal muscle properties, and colonic genes expressions for control and antibiotic-treated piglets were tested for significance with the Student’s *t*-test using statistic software SAS 9.1 (SAS Institute Inc., NC, USA), and *P* values lower than 0.05 were considered as statistically significant. For the bacterial data, the indices of alpha diversity and the relative abundances of taxa were compared with the non-parametric Kruskal-Wallis tests, and the intragroup statistic differences in beta diversity were assessed using permutational multivariate analysis of variance (PERMANOVA) with 999 permutations. Corrections for the multiple comparisons in statistic testing were made using the Benjamini Hochberg method. The differences were considered significant when the adjusted *P* values < 0.05.

## Results

### No significant differences were observed in initial fecal microbiota composition between groups

**Fig. 1:**
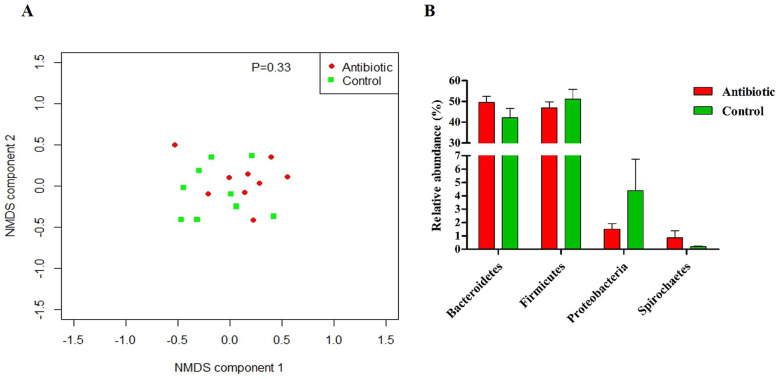
Initial fecal microbiota composition of piglets before antibiotic treatment. **a**. NMDS plot, based on the Bray-Curtis dissimilarity indices, displays the beta diversity in both groups. **b**. The differences in four predominant phyla between antibiotic-treated piglets and control piglets. *P < 0.05, ** *P* < 0.01. *n* = 9 for each group

Since the initial gut microbiota composition may influence the host response to antibiotic exposure [28], the initial fecal microbiota composition of two groups were determined by 16S rRNA gene amplicon sequencing. The antibiotic and control group exhibited similar alpha diversity, as there were no differences in richness (Chao1 index), diversity (Shannon index) and evenness (InvSimpson index) (Additional file Table S3). The non-metric multidimensional scaling (NMDS) plot that visualized the Bray-Curtis dissimilarity index of the samples revealed no significant difference in beta diversity of fecal microbiota community between the two groups (Fig. 1a). No significant differences were prevalent in the relative abundance of *Firmicutes*, *Bacteroidetes*, *Proteobacteria* and *Spirochaetes* between the groups (Fig. 1b). The relative abundance for class, order and family levels are described in Supplemental results (Additional File S2, Fig. S1, S2, S3). At the genus level, none of the OTUs were significantly differentiated by groups (Additional file Fig. S4). The high similarity in microbiota composition between the groups indicates that the initial fecal microbiota was likely not to interfere with the outcomes from antibiotic exposure.

### Effects of antibiotic exposure on growth rate and carcass composition of piglets

**Fig. 2:**
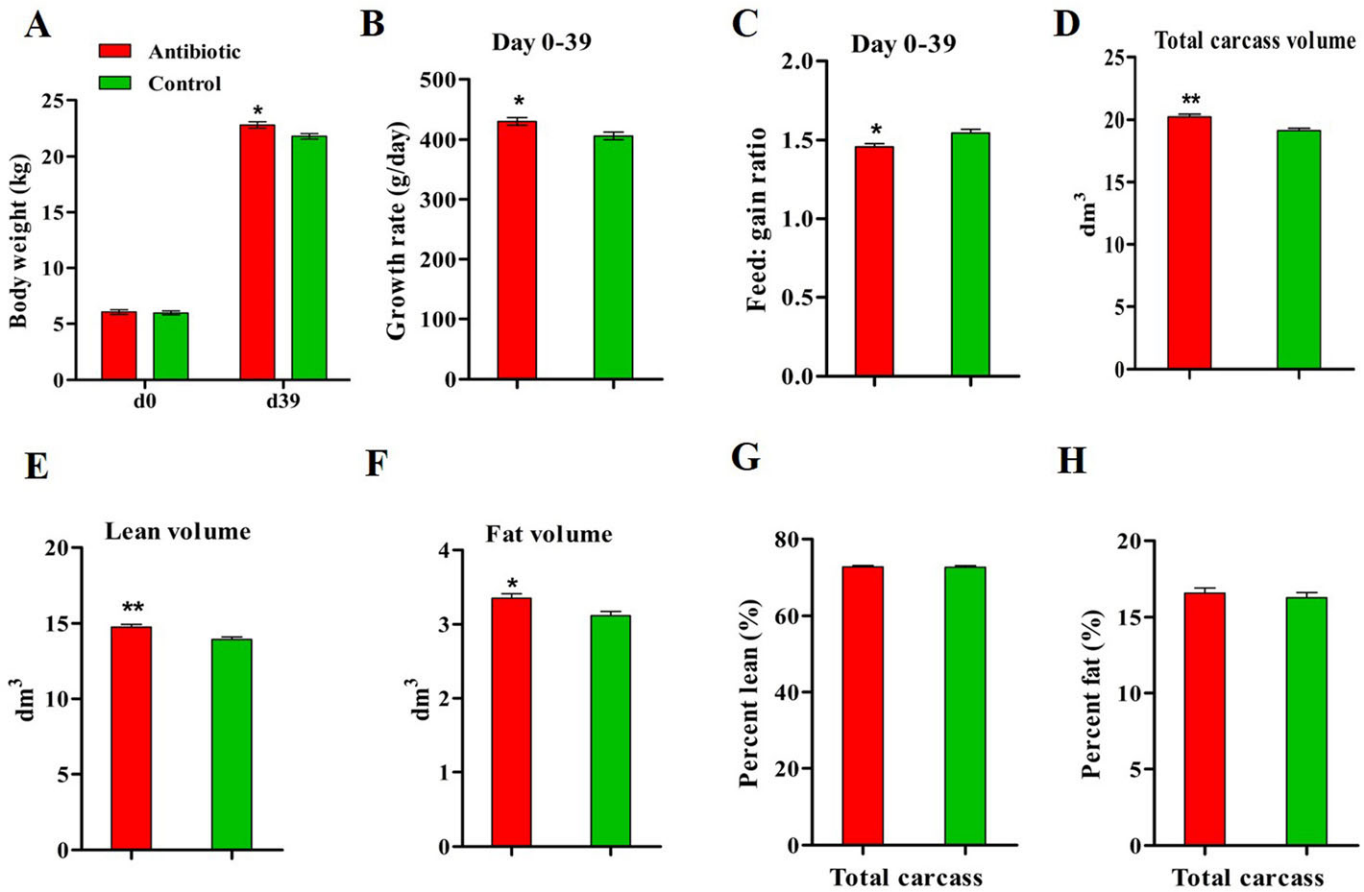
Effects of antibiotic exposure on growth and carcass composition of piglets. **a-c**. Body weight after 39 days of antibiotic administration and growth rate and feed: gain ratio in the period d0-d39. **d-h**. Carcass composition assessed at d39 by computed tomography scanning. *P < 0.05, ** *P* < 0.01. *n* = 9 for each group

Weaning piglets were fed with tylosin phosphate at a dosage in the range of approved levels according to the US Food and Drug Administration (FDA) for using as an antimicrobial growth promotor in livestock production. After 39 days’ exposure, an effect was observed in terms of body weight and growth rate, with antibiotic-treated piglets growing faster than control piglets (Fig. 2a and b) (*P* < 0.05). Antibiotic administration improved the feed conversion efficiency of the piglets (Fig. 2c) (*P* < 0.05). The distribution of volumes based on Hounsfield unit (HU) values indicated that the fat and lean peak value were increased by antibiotic exposure (Additional file Fig. S5). Antibiotic administration increased the total volume, lean volume and fat volume in the carcass of piglets (Fig. 2d, e and f) (*P* < 0.05), but the lean and fat percentages were not different between treatments (Fig. 2g and h). Hence, antibiotic administration concurrently accelerated the lean and fat deposition in the porcine carcass.

### Effects of antibiotic exposure on serum lipid profile and muscle fat content of piglets

**Fig. 3:**
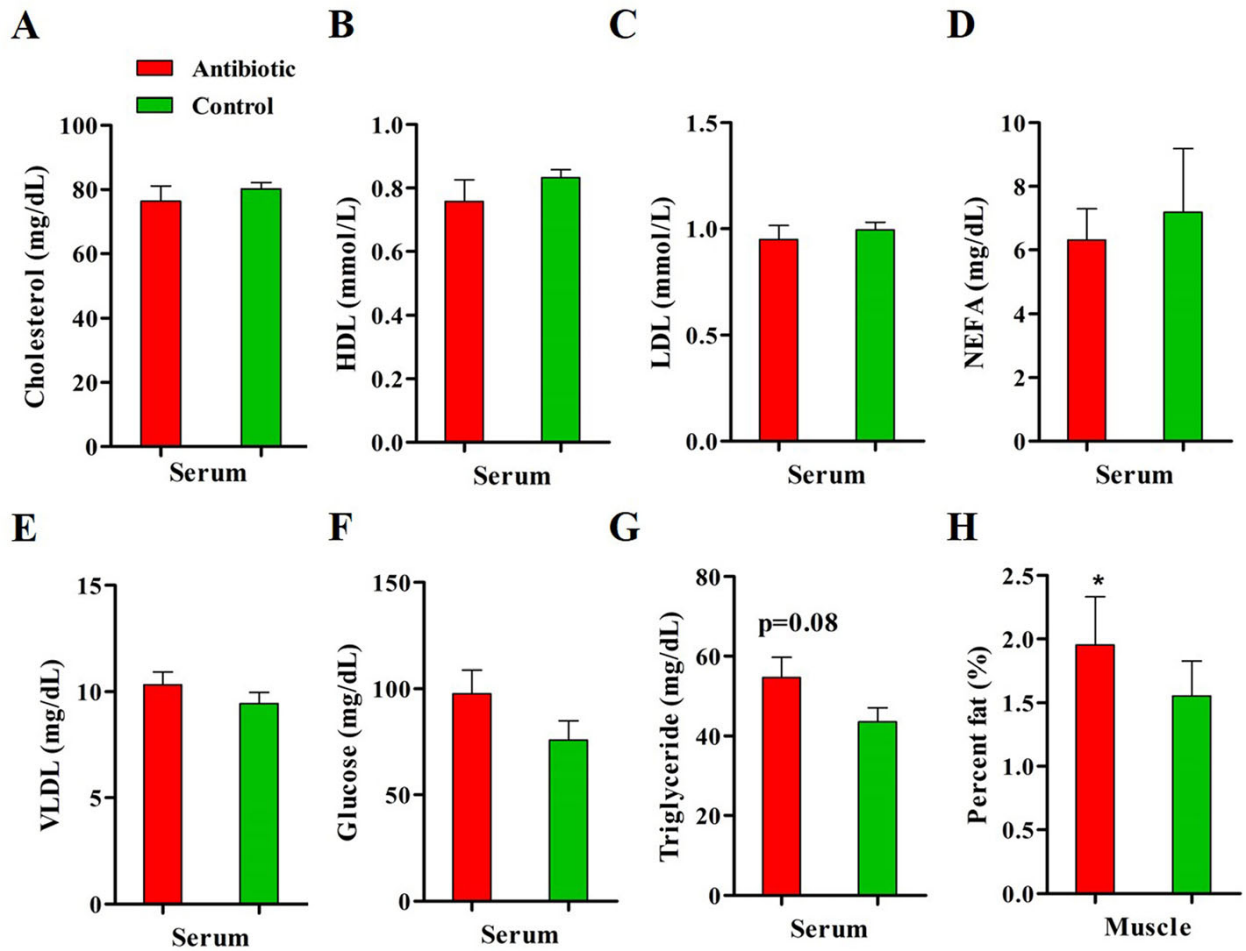
Serum metabolites level and intramuscular fat content after antibiotic exposure. **a-g**. Serum lipid profile and glucose level at the end of the experiment determined by spectrophotometry. **h**. Intramuscular fat content in longissimus muscle measured by Soxhlet extraction. HDL, high density lipoprotein; LDL, low density lipoprotein; NEFA, non-esterified fatty acid; VLDL, very-low density lipoprotein. *P < 0.05, ** *P* < 0.01. *n* = 9 for each group

No differences in serum cholesterol, HDL, LDL, VLDL, NEFA and glucose levels were observed between groups. The serum triglyceride concentration tended to increase in the antibiotic-treated piglets as compared to controls (*P* = 0.08) (Fig. 3g). The fat deposition in muscle was increased by dietary antibiotic intake, as indicated by higher IMF content in *longissimus* muscle in antibiotic-treated piglets compared to controls (*P* < 0.05) (Fig. 3h).

### Antibiotic exposure altered muscle fiber characteristics and composition of piglets

**Fig. 4:**
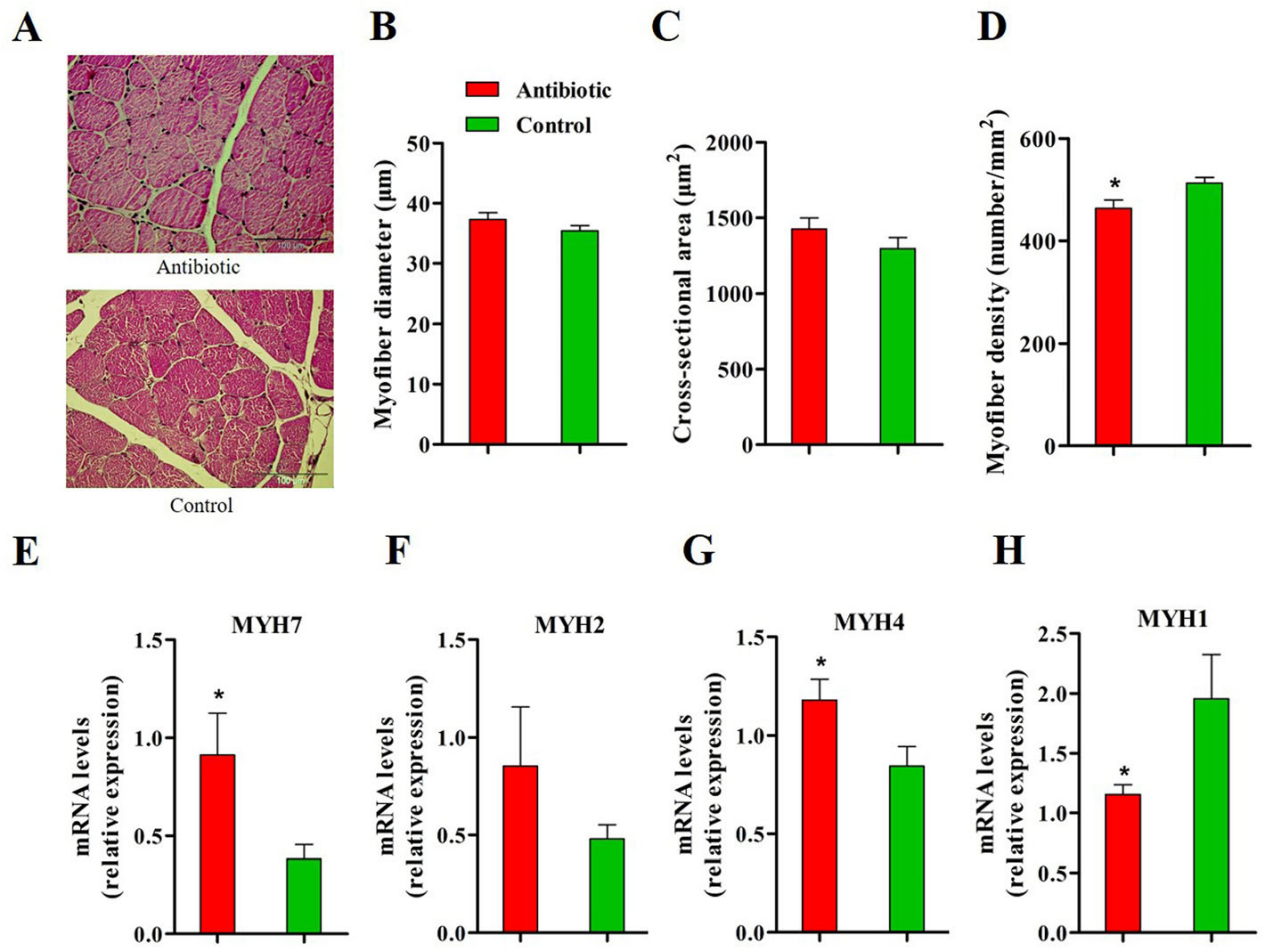
Antibiotic exposure alters myofiber characteristics and types composition in *longissimus* muscle of piglets. **a**. Histological images of muscle obtained from H&E staining with no apparent difference in myofiber size between groups. **b-d**. Myofiber diameter, cross-sectional area and density. **e-h**. Myosin heavy chain isoforms expression in muscle. MYH, myosin heavy chain. *P < 0.05, ** *P* < 0.01. *n* = 9 for each group

No visible differences between groups in muscle morphology were observed on representative images (Fig. 4a). The processed results showed no significant treatment effect on myofiber diameter and cross-sectional area (Fig. 4b and c), whereas antibiotic exposure decreased the myofiber density in *longissimus* muscle (Fig. 4d, *P* < 0.05). The antibiotic treatment up-regulated the muscular mRNA expression of *MYH7* and *MYH4*, which encode the slow-contracting fiber and fast IIb fiber, respectively (Fig. 4e and g) (*P* < 0.05). Antibiotic-treated piglets exhibited lower mRNA expression of *MYH1*, which encodes the fast IIx fiber, as compared to controls (Fig. 4h) (*P* < 0.05).

### Antibiotic exposure affected the expression of lipid metabolism-related genes

**Fig. 5:**
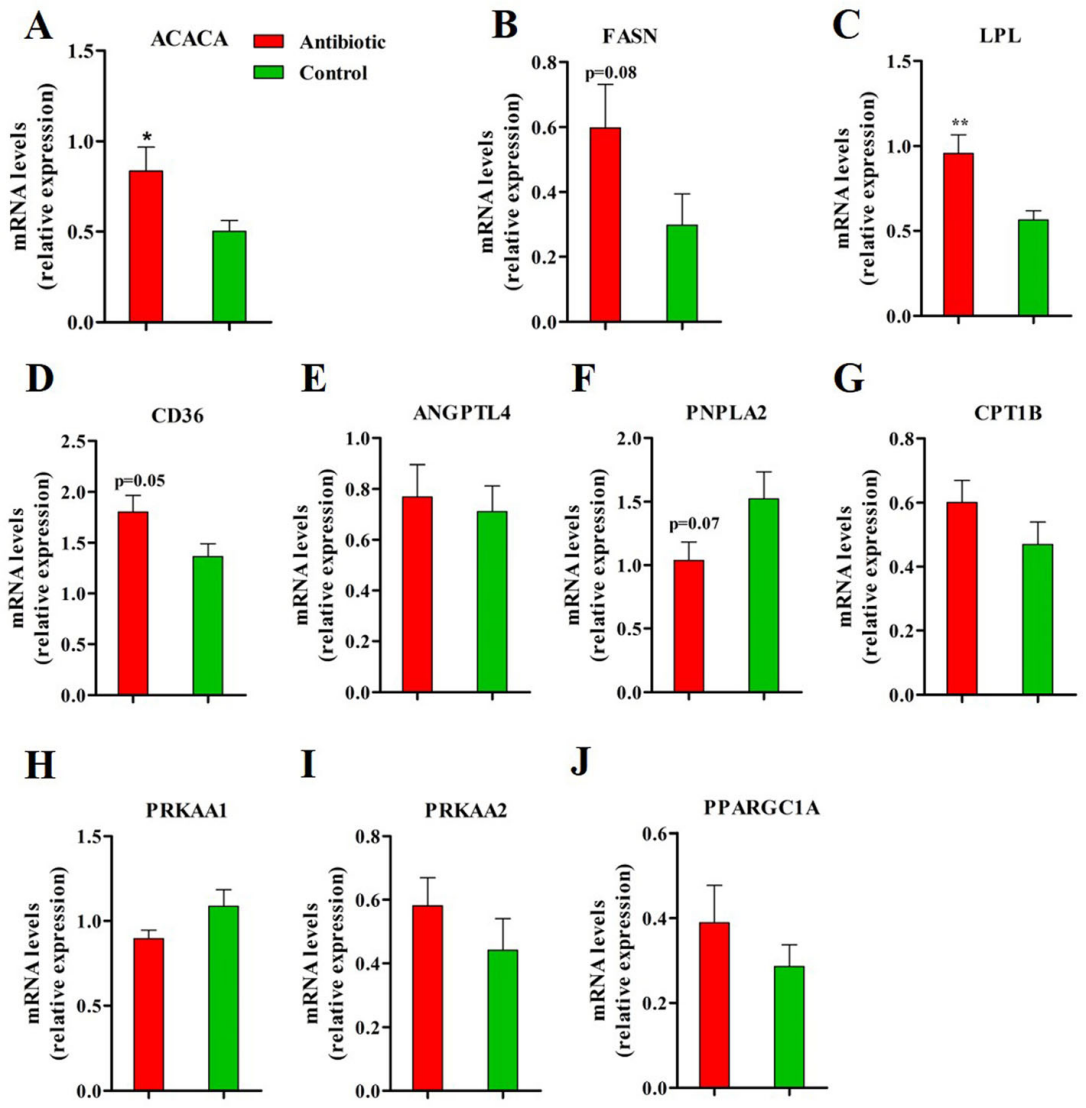
Lipid metabolism-related genes expression in *longissimus* muscle determined through quantitative PCR analyses. ACACA, acetyl-CoA carboxylase alpha; FASN, fatty acid synthase; LPL, lipoprotein lipase; CD36, CD36 molecule; ANGPTL4, angiopoietin like 4; PNPLA2, patatin-like phospholipase domain containing 2; CPT1B, carnitine palmitoyl transferase 1B; PRKAA1, protein kinase AMP-activated catalytic subunit alpha 1; PRKAA2, protein kinase AMP-activated catalytic subunit alpha 2; PPARGC1A, PPARG coactivator 1 alpha. *P < 0.05, ** *P* < 0.01. *n* = 9 for each group

To investigate how the in-feed antibiotic affects the lipid deposition in muscle per se, the expression of metabolic genes in *longissimus* muscle from piglets fed either a tylosin-containing or control diet were analyzed. An increased expression of *ACACA* (*P* < 0.05) and a trend towards higher mRNA abundance of *FASN* (*P* = 0.08) were observed in antibiotic-treated versus control piglets (Fig. 5a and b). Antibiotic administration enhanced the muscular expression of *LPL* (*P* < 0.05) and *CD36* (*P* = 0.05), (Fig. 5c and d). The expression of angiopoietin like 4 (*ANGPTL4*), which acts as an inhibitor of *LPL* transcription and enzyme activity, was not influenced by antibiotic exposure (Fig. 5e). *PNPLA2* expression tended to decrease in antibiotic-treated piglets compared to controls (Fig. 5f, *P* = 0.07). No treatment effects were observed on the muscular expression of *CPT1B* (Fig. 5g) and its upstream molecules protein kinase AMP-activated catalytic subunit alpha 1 and alpha 2 (*PRKAA1* and *PRKAA2*) (Fig. 5h and i). No significant differences were observed between groups for the mRNA level of peroxisome proliferator activated receptor gamma (*PPARG*) coactivator 1 alpha (*PPARGC1A*) (Fig. 5j).

### Antibiotic exposure resulted in an altered gut microbiota

**Fig. 6:**
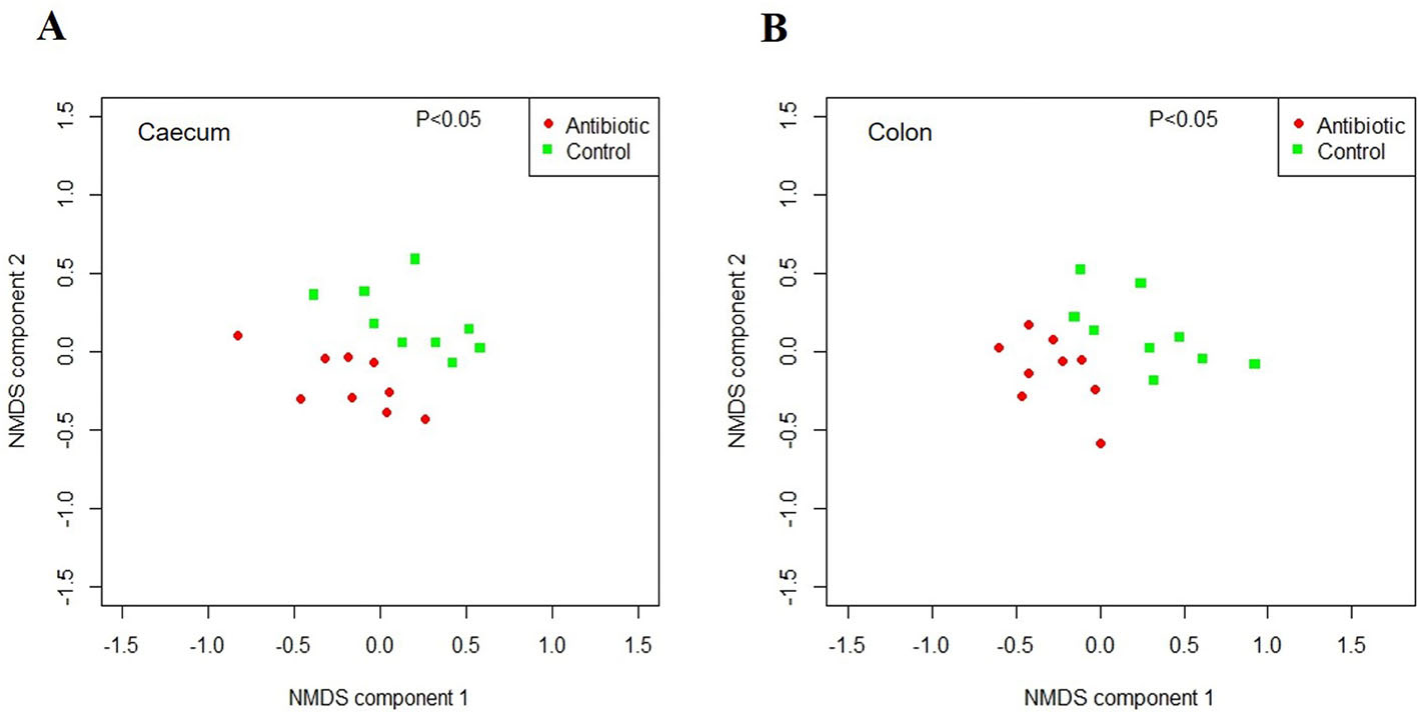
NMDS plots, based on the Bray-Curtis dissimilarity indices, display the beta diversity of caecal **a** and colonic microbiota **b** between groups. *n* = 9 for each group

**Fig. 7:**
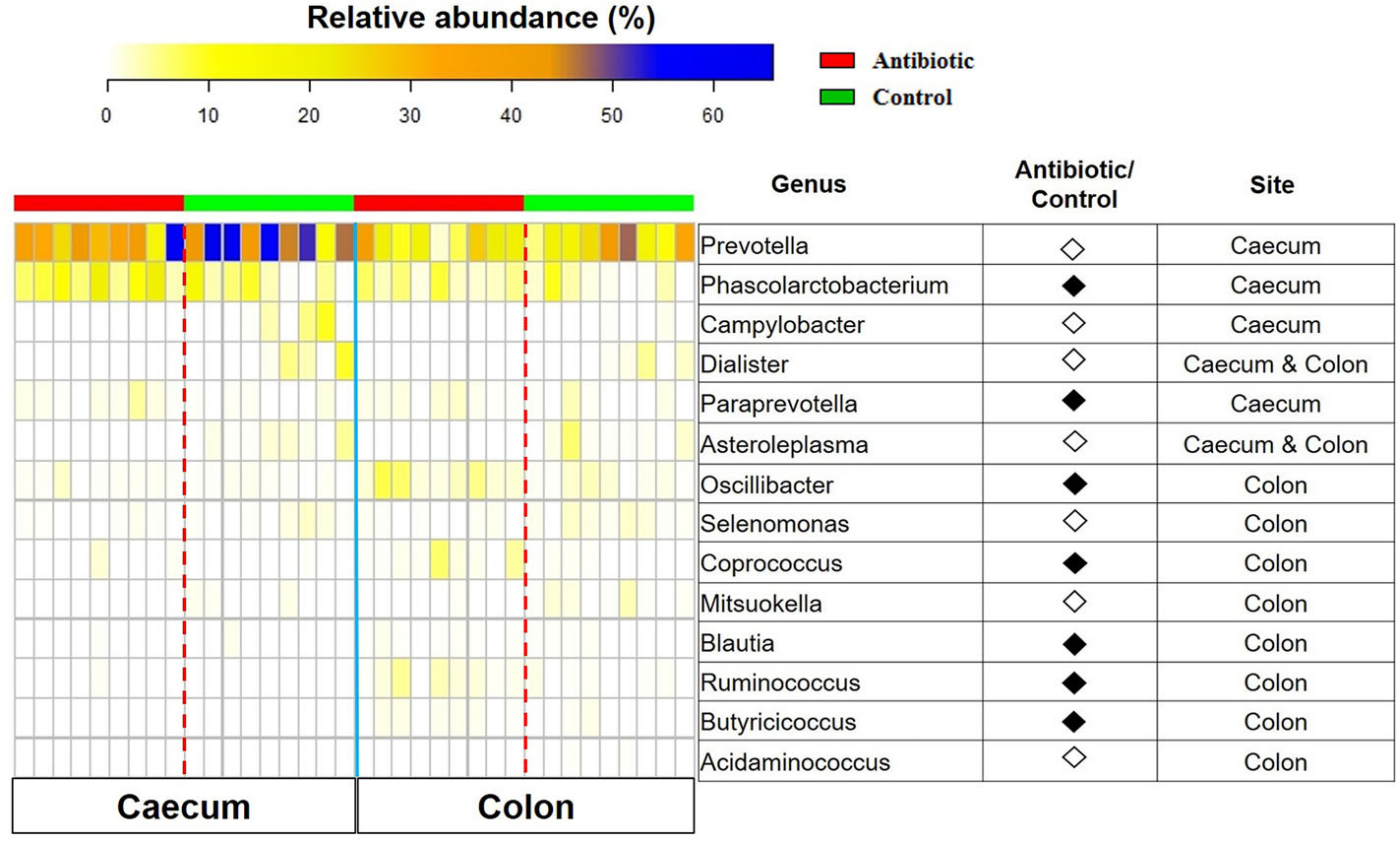
Heatmap displays the differentially abundant genera between antibiotic and control group. The intestinal sites in which significant differences appear are indicated in the panel. The closed black diamonds represent that the column numerator has higher abundance of the genus than denominator. The hollow diamonds represent that the column numerator has lower abundance of the genus than denominator (Adjusted *p* value lower than 0.05). *n* = 9 for each group

No significant differences in the alpha diversity indices were observed in both caecum and colon microbiota between the groups (Additional file Table S3). On the other hand, the NMDS plots of the Bray Curtis distance showed significant separate clustering of samples according to groups (Fig. 6a and b). At the phylum level, *Firmicutes* was increased in the caecum (*P* < 0.05) and tended to increase in the colon (*P* = 0.069) of antibiotic-treated piglets, while *Bacteroidetes* tended to increase in the caecum (*P* = 0.056) and colon (*P* = 0.076) of the control group (Additional file Fig. S6A and 6B). The ratio of relative abundance of *Firmicutes* and *Bacteroidetes* (F/B ratio) was increased in the caecum of antibiotic-treated pigs compared to controls (Additional file Fig. S6A) (*P* < 0.05). Following the antibiotic treatment, a decreased abundance of *Tenericutes* was observed in the caecum (*P* < 0.05) and a trend towards a decrease in the colon (*P* = 0.069) (Additional file Fig. S6A and 6B). At the genus level, *Dialister* and *Asteroleplasma* were decreased in caecum and colon of antibiotic-treated piglets, *Prevotella* and *Campylobacter* were decreased in the caecum, and *Selenomonas*, *Misuokella*, and *Acidaminococcus* were decreased in the colon of piglets exposed to antibiotic. Antibiotic exposure increased the abundance of *Phascolarctobacterium* and *Paraprevotella* in the caecum, and *Oscillibacter*, *Coprococcus*, *Blautia*, *Ruminococcus*, and *Butyricicoccus* in the colon (Fig. 7) (*P* < 0.05). This suggests that antibiotic administration results in an altered gut microbiota composition in the hindgut of piglets.

## Discussion

Studies focusing on the link between gut microbiota and host physiology have revealed the determinant role of intestinal microbiota in modulating host lipid metabolism [2, 3]. Skeletal muscles are important sites for the utilization and storage of lipids, and as our previous study provided evidence that the gut microbiota shapes the myofiber composition and lipid metabolism of skeletal muscle [7], here, we evaluated the effects of antibiotic administration on gut microbiota and skeletal muscle properties. We adopted a piglet model of post-weaning antibiotic treatment to understand the consequences of a sub-therapeutic dosage (40 to 100 mg/kg of diet) of tylosin phosphate administration on gut microbiota composition and host metabolism.

Host responses to antibiotic treatment were different between mice harboring the initial gut microbiota with and without *Escherichia coli*, indicating that slight differences in initial gut microbiota might influence the consequences of antibiotic exposure [28]. Piglets used in the present study were assigned to the treatments by balancing body weight as well as litter, because the genetic background and body weight were found to be closely associated with the microbiota profile [29, 30]. We compared the fecal microbiota composition between groups before antibiotic treatment, and found no significant differences. The highly similar microbiota at the beginning of the trial excludes possible confounding of the initial microbiota on alterations of the bacterial population and the host physiology due to the antibiotic treatment.

Although tylosin addition in feed has been used as a method to accelerate growth performance of pigs, previous studies involving tylosin treatment, differing in design, yielded conflicting results [31, 32]. In our experiment, an increased growth rate and feed conversion efficiency were observed in antibiotic-treated piglets, indicating that tylosin-treated piglets exhibited a more efficient digestion and utilization of nutrients [33]. Previous studies demonstrated that antibiotic-treated pigs had higher carcass lean percentage than untreated ones [31, 34]. However, antibiotic exposure induced a gain in fat mass in mice [9, 10, 17]. Unlike these observations, we found that antibiotic treatment increased both lean volume and fat volume of the carcasses, whereas the lean and fat proportions of the carcasses did not differ between the groups. The diet composition, animal genetic background and antibiotic dosage may contribute to these inconsistent effects.

In agreement with the findings of a recent study [33], we found that antibiotic exposure elevated the serum triglyceride concentration. In terms of myofiber characteristics, the absolute value of myofiber diameter and cross-sectional area were higher in *longissimus* muscle of antibiotic-treated piglets, and the myofiber density was significantly decreased by antibiotic exposure. In line with other studies, myofiber diameter and cross-sectional area were positively correlated with growth rate and feed conversion efficiency [35, 36]. This suggests that tylosin administration increased muscle growth in the present study. Carcass weight has been associated with myofiber composition, as indicated by higher type IIb fibers and lower type I and IIa fibers in pigs with heavier carcasses [37]. However, we observed that antibiotic exposure increased mRNA levels of myosin isoforms determining type I and IIb fibers, but decreased type IIx fibers at the transcriptional level in the *longissimus* muscle of piglets. The nuclear receptor coactivator PPARGC1A has been shown to play a pivotal role in regulating slow, oxidative myofiber development, as indicated by reduced type I fibers in PPARGC1A knockout mice, compared to their wild-type counterparts [38]. In the current study, no difference at the transcription level of PPARGC1A in *longissimus* muscle was observed between groups, suggesting that the PPARGC1A may not be the principal factor in determining the type I fibers content in skeletal muscle of meat-producing animals [39]. Although a causal relationship between body composition and myofiber composition remains unclear, close associations have been revealed previously [7, 40]. The joint promotion in carcass muscle growth and fat deposition, as observed in our study, might be the cause or the consequence of the shift in fiber-type composition.

We found that post-weaning tylosin ingestion elevated the IMF content in the *longissimus* muscle of piglets. The enhanced fat deposition occurred in non-adipose tissues, like liver and skeletal muscle, in which the uptake of free fatty acids was increased, whereas the fatty acid oxidation was decreased [41]. In the present study, the LPL and CD36 abundance were up-regulated in the *longissimus* muscle of antibiotic-treated piglets, indicating that more fatty acids were imported into the skeletal muscle [42]. Higher ACACA were observed in piglets exposed to antibiotics, indicative of enhanced de novo fatty acid synthesis in skeletal muscle [41]. However, no differences in gene expressions of CPT1B, PRKAA1 and PRKAA2 were observed, demonstrating that the fatty acid oxidation in skeletal muscle was not affected by antibiotic exposure [41]. The LPL inhibitor ANGPTL4 [43] has been shown to regulate LPL transcription and activity in peripheral tissues by its circulating level instead of the expression in these tissues per se [2]. In agreement, no significant dietary effect on ANGPTL4 gene expression in skeletal muscle was observed in our study. These findings suggest that the increased fat deposition in skeletal muscle of piglets exposed to antibiotic is metabolically altered through increased fatty acid uptake and synthesis as well as decreased triglyceride hydrolysis without affecting fatty acid oxidation.

Gut microbiota have been shown to mediate the actions of antibiotic treatment on growth and metabolism of animals [11–13]. We have also identified the key microbiota characteristics in tylosin-treated piglets. First, consistent with a recent study [32], tylosin exposure did not alter the alpha diversity of the caecum and colon microbiota. Second, although differences in tylosin-perturbed taxonomic distributions were apparent between intestinal locations [44], the significant clustering of samples by groups occurred in both caecum and colon. Taxa were identified that may be associated with the antibiotic-caused alterations in growth and skeletal muscle properties of piglets. Although the relationship between the relative abundance of *Firmicutes* and *Bacteroidetes* as well as the F/B ratio and body composition is inconclusive [45, 46], our results demonstrated a higher *Firmicutes* abundance and F/B ratio as well as lower *Bacteroidetes* abundance in the tylosin-treated piglets [32]. The *Tenericutes* phylum was previously reported to be positively correlated with crude fiber (CF) digestibility of pigs [47]. We observed that tylosin exposure decreased the relative abundance of *Tenericutes*, which was in line with another study in which faster growing pigs exhibited lower CF digestibility than slower ones [48].

The microbiota composition at genus level has been closely correlated with host growth traits and metabolism in several studies [49–51]. A recent study establishing the links between growth traits and gut microbiota composition has shown a higher abundance of genera *Prevotella* and *Mitsuokella* and lower abundance of *Ruminococcus* in piglets with high body weight and growth rate compared to counterparts [51]. However, we got completely opposite results, suggesting that a different gut microbiota response may exist between spontaneous and antibiotic-induced growth promotion. The *Prevotella and Ruminococcus* genera were reported to be lower and higher, respectively, in obese pigs with low growth rate and obese mice [1, 7]. Antibiotic exposure decreased the *Prevotella* proportion in the colon of non-obese diabetic mice [52], indicating that the host metabolic phenotypes should be considered in building up a correlation between microbiota composition and growth traits. An epidemiologic study has shown that patients with non-alcoholic fatty liver disease exhibit lower *Prevotella* abundance than healthy subjects [53], and rats fed with high-fat high-sucrose diet had higher intramuscular fat and a lower abundance of the genus *Prevotella* [54], suggesting the increased ectopic fat deposition in liver or skeletal muscle may be associated with lower *Prevotella* abundance. We found that tylosin exposure increased the relative abundance of *Paraprevotella*, *Coprococcus*, and *Oscillibacter*, which were also higher in antibiotic-treated pigs or pigs with high feed efficiency in other studies [44, 50, 55]. The relative abundance of genus *Blautia* was higher in the colon of tylosin-treated piglets, which was consistent with our previous study, showing that pigs with higher IMF content exhibited increased *Blautia* proportions in the feces [7]. Previous studies showed that the relative abundance of *Phascolarctobacterium* were increased in mice exposed to a high-fat diet, and it was found to be positively correlated with serum triglyceride level [56, 57]. Consistently, we also observed higher serum triglyceride level and increased abundance of *Phascolarctobacterium* in caecum of antibiotic-treated piglets. Antibiotic exposure has been shown to increase the succinate accumulation in the gut, which promote the growth of succinate-consuming bacteria *Phascolarctobacterium* [58, 59]. This suggests that tylosin administration causes alterations in the gut microbiota composition, which is associated with improvements in growth performance and alterations in muscular metabolism of piglets.

## Conclusions

Our results demonstrated that tylosin-treated piglets (1) had higher growth rate and feed efficiency; (2) exhibited altered myofiber characteristics and composition; (3) had increased IMF deposition, which was metabolically altered; (4) had an altered caecum and colon microbiota composition, as well as specific genes expressions in colon, which were associated with the outlined alterations in the host phenotype. This confirms that modulating the gut microbiota is a potential approach to influence the myofiber development and IMF content of pigs, and that dietary intervention targeting the gut microbiota to regulate skeletal muscle properties is possible.

## Supplementary information

**Additional file 1.**

## Data Availability

The raw data files of the sequences processed in this study have been deposited in the National Center for Biotechnology Information (NCBI) database (Accession number SRP130427).

## Abbreviations

*ACTB*: β-actin
*TOP2B*: Topoisomerase (DNA) IIb
*TBP*: TATA box binding protein
*YWHAZ*: Tyrosine 3-monooxygenase/tryptophan 5-monooxygenase activation protein
*ACACA*: Acetyl-CoA carboxylase alpha
*FASN*: Fatty acid synthase
*CPT1B*: Carnitine palmitoyl-transferase 1B
*LPL*: Lipoprotein lipase
*CD36*: CD36 molecule
*PNPLA2*: Patatin-like phospholipase domain containing 2, also known as ATGL
*PRKAA1*: Protein kinase AMP-activated catalytic subunit alpha 1
PRKAA2: Protein kinase AMP-activated catalytic subunit alpha 2
*PPARGC1A*: Peroxisome proliferator activated receptor gamma coactivator 1 alpha
*MYH7*: Myosin heavy chain 7
*MYH2*: Myosin heavy chain 2
*MYH4*: Myosin heavy chain 4
*MYH1*: Myosin heavy chain 1
