# Supplementary material for: Antibiotic affects the gut microbiota composition and expression of genes related to lipid metabolism and myofiber types in skeletal muscle of piglets
Source: BMC Vet Res. 2020 Oct 16;16:392. doi: 10.1186/s12917-020-02592-0 (PMC7568366; doi:10.1186/s12917-020-02592-0)
Supplement: Supplementary file 1 — Additional file 1. [file 12917_2020_2592_MOESM1_ESM.docx]

**Antibiotic affects the gut microbiota composition and expression of genes related to lipid metabolism and myofiber types in skeletal muscle of piglets**

Honglin Yan^1,2,3^, Bing Yu^1^, Jeroen Degroote^2^, Thomas Spranghers^2^, Noémie Van Noten^2^, Maryam Majdeddin^2^, Mario Van Poucke^4^, Luc Peelman^4^, Jo De Vrieze^5^, Nico Boon^5^, Ingrid Gielen^6^, Stefaan De Smet^2*^, Daiwen Chen^1*^, Joris Michiels^2^

^1^Animal Nutrition Institute, Sichuan Agricultural University, Key Laboratory of Animal Disease-Resistance Nutrition, Ministry of Education, China, Ya’an 625014, People’s Republic of China.

^2^Laboratory for Animal Nutrition and Animal Product Quality, Department of Animal Sciences and Aquatic Ecology, Ghent University, Coupure Links 653, 9000 Ghent, Belgium.

^3^School of Life Science and Engineering, Southwest University of Science and Technology, Mianyang 621010, People’s Republic of China

^4^Department of Nutrition, Genetics and Ethology, Ghent University, Heidestraat 19, 9820 Merelbeke, Belgium.

^5^Center for Microbial Ecology and Technology (CMET), Ghent University, Coupure Links 653, 9000, Ghent, Belgium.

^6^Department of Medical Imaging and Small Animal Orthopaedics, Ghent University, Salisburylaan 133, 9820 Merelbeke, Belgium

**^*^Correspondence:** Daiwen Chen, E-mail address: [dwchen@sicau.edu.cn](mailto:dwchen@sicau.edu.cn).

# Contents

[Contents 2](#_Toc494648290)

[S1 Supplemental experimental procedures 3](#_Toc494648291)

[S1.1 Computed tomography scanning of carcasses 3](#_Toc494648292)

[S1.2 RNA extraction, cDNA synthesis and primers picking 3](#_Toc494648293)

[S1.3 Illumina data processing 5](#_Toc494648294)

[S2 Supplemental results 5](#_Toc494648295)

[S3 Supplemental tables 6](#_Toc494648296)

[S4 Supplemental figures 9](#_Toc494648297)

[References 16](#_Toc494648298)

# S1 Supplemental experimental procedures

## S1.1 Computed tomography scanning of carcasses

After all samples were collected, the genital organs, diaphragm, flare fat and viscera were removed, and the remaining carcass was flushed and hanged by the hoof on a steel hook for draining water, followed by cooling at 4 °C pending scanning. Within 24 h post-mortem, carcasses were scanned with a GE Lightspeed Qx/I scanner. Scanning was done following the protocol described by Font i Furnols et al. [[1](#_ENREF_1)] with some modifications: 120 kV and 180 mA, 512 × 512 matrix and 4-mm slices thickness. The “standard” reconstruction algorithm was used to create transversal images, and the Matlab software 9.0 was used to analyze the images, and calculate lean volume, fat volume and bone volume of the carcass based on the Hounsfield scale. The estimated volumes in the Hounsfield units (HU) range [ -149, -1], HU range [0, 140] and HU range [141, 1000] were associated with fat, muscle and bone volumes, respectively [[2](#_ENREF_2)]. The sampled muscle weight was converted to volume by dividing by the muscle density, as determined earlier [[3](#_ENREF_3)]. The sampled muscle volume was used to correct the carcass lean volume of each piglet. The total volume of the carcass was the sum of three partial volumes, and the lean and fat percentage were obtained by dividing the calibrated lean volume and fat volume by total volume.

## S1.2 RNA extraction, cDNA synthesis and primers picking

Total RNA from *longissimus* muscle and colon mucosa were extracted using Bio-Rad Aurum Total RNA Fatty and Fibrous Tissue Kit (Bio-Rad Laboratories, Hercules, CA, USA) which included a genomic DNA removal step by on-column DNase treatment. Before synthesizing the cDNA, RNA quality control was performed. The purity (OD 260/280, ranging from 1.9 to 2.2, and OD 260/230, ranging from 2.0 to 2.2) and concentration of total RNA (ranging from 150 to 1200 ng/μl) were measured using the NanoDrop ND-1000 (Nanodrop Technologies, Thermo Scientific, Wilmington, DE, USA). 1 μg of RNA was loaded on a 2% agarose gel to verify the integrity by visually evaluating the 28S and 18S ribosomal RNA bands, and the verification of the elimination of genomic DNA in the isolated RNA was done by the minus reverse transcription control PCR using YWHAZ primers, as previously described [[4](#_ENREF_4)]. The cDNA was synthesized *via* reverse transcription, which was performed with 1 μg of high-quality gDNA-free RNA in a 20 μL reaction system using the ImProm-II cDNA synthesis kit (Promega, Madison, WI, USA). The synthesized cDNA was 10× diluted by adding 180 μL Milli-Q water, and the verification of cDNA was done by a control PCR using 2 μL diluted cDNA and the YWHAZ primers. Primers used for most target genes (MYH7, MYH2, MYH4, MYH1, ACACA, FASN, LPL, CD36, PNPLA2, CPT1, PRKAA1, PRKAA2) were designed using the NCBI Primer-Blast Tool (<http://www.ncbi.nlm.nih.gov/tools/primer-blast/>) [[5](#_ENREF_5)], based on the certain exon-exon boundaries of published gene sequences of pigs. The single nucleotide polymorphism and secondary structure in target sequences were checked with dbSNP database and mFold [[6](#_ENREF_6)], respectively. The primers of PPARGC1A, YWHAZ, TBP, TOP2B and ACTIN were obtained from Erkens et al [[4](#_ENREF_4)].

## S1.3 Illumina data processing

After the completion of sequencing, reads with a quality score < 20 were filtered using Sickle v1.33 [7], the error correction of reads was done using BayesHammer together with Spades v2.5.0 [8], and the reads with a minimum overlap of 20 bases were assembled by PANDAseq v2.4 [9]. The quality-filtered reads were pre-processed through the Mothur software [[10](#_ENREF_11)] involving: (1) clustering the reads into operational taxonomical units (OTUs) using the USEARCH v7.0.1001 [[11](#_ENREF_12)] based on 97% similarity and the chimera OTUs were filtered using UCHIME v4.2 [[12](#_ENREF_13)]; (2) the representative sequence from each OTU-cluster was picked and used for taxonomic assignment of each OTU against the Ribosomal Database Project (RDP) database with the RDP v2.6 [[13](#_ENREF_14)].

# S2 Supplemental results

At the class level, *Bacteroidia* was the most abundant taxon in both antibiotic group (ANT, 45.00%) and control group (CON, 40.06%), and the next three abundant classes were *Clostridia* (29.08% and 23.04% in ANT and CON, respectively), *Negativicutes* (10.06% and 18.88%) and *Gammaproteobacteria* (0.56% and 3.86%), but there were no significant differences between treatments (Fig. S1). The predominant orders in the fecal microbiota from piglets were *Bacteroidales*, *Clostridiales*, *Lactobacillales*, *Selenomonadales* (*Firmicutes*) and *Enterobacteriales* (*Proteobacteria*), and no significant differences were observed between ANT and CON (Fig. S2). At the family level, no significant group differences were detected regarding the high abundant families (>3%) *Acidaminococcaceae*, *Lachnospiraceae*, *Lactobacillaceae*, *Porphyromonadaceae*, *Prevotellaceae*, *Ruminococcaceae* and *Veillonellaceae* (Fig. S3)

# S3 Supplemental tables

Table S1. Feed ingredients and nutrient content of the diet of the piglets

| Ingredients, % | content | Nutrient level ^a^ | content |
| --- | --- | --- | --- |
| Barley | 20.000 | Dry matter, % | 88.680 |
| Corn | 24.256 | Crude protein, % | 18.000 |
| Toasted soybeans | 16.000 | Crude fat, % | 5.450 |
| Wheat | 12.000 | Starch, % | 36.850 |
| Oat flakes | 8.000 | Sugars, % | 7.970 |
| Sweet wheypowder | 6.000 | Sugars+starch, % | 44.820 |
| Soybean meal cp48 | 4.657 | Ash, % | 5.310 |
| Wheat gluten protein cp82 | 2.000 | Crude fibre, % | 4.070 |
| Premix trace min & vit ^b^ | 1.000 | Calcium, % | 0.660 |
| Potato protein | 1.000 | Digestible P, % | 0.310 |
| Sugar beet pulp | 1.000 | EB (Na+K-CL; meq/100g) | 195.0 |
| Monocalciumphosphate | 0.946 | AID Lys, % | 1.100 |
| Limestone | 0.929 | AID Met, % | 0.418 |
| L-lysine-HCL | 0.534 | AID Met+Cys, % | 0.649 |
| Lactose | 0.513 | AID Trp, % | 0.231 |
| Sodiumbicarbonate | 0.370 | AID thr, % | 0.682 |
| Salt | 0.240 | AID Val, % | 0.748 |
| L-Threonine | 0.194 | AID Ile, % | 0.583 |
| DL-Methionine | 0.188 | NE, Kcal/kg | 2385 |
| L-Valine | 0.102 | ME, Kcal/kg | 3095 |
| L-Tryptophan | 0.071 |  |  |
| Sum | 100 |  |  |

^a^ All data are calculated values.

^b^ Providing per kg of diet: vit A (retinyl acetate), 15000 IU; vit D3 (cholecalciferol), 2000 IU; vit E (all-rac-alfa-tocopherylacetate), 50.0 mg; vit K3 (menadion), 4.0 mg; vit B1 (thiamine mononitrate), 3.1 mg; vit B2 (riboflavine), 8.0 mg; vit B3 (calcium-D-pantothenate), 20 mg; vit B6 (pyridoxine hydrochloride), 6.0 mg; vit B12 (cyanocobalamine), 50.0 µg; vit PP (niacinamide), 40.0 mg; folic acid, 2.0 mg; biotin, 0.3 mg; betaine anhydrate, 285 mg; endo-1,4-beta-glucanase E3.2.1.4, 250 TGU; endo-1,4-beta-xylanase E3.2.1.8, 560 TXU; Cu (copper(II)sulphate pentahydrate), 15.0 mg; Zn (zinc oxide), 100 mg; Mn (manganese(II)oxide), 48.0 mg; I (calciumjodate anhydrate), 1.9 mg; Se (sodium selenite), 200 µg; Se (selenomethionine produced by *Saccharomyces cerevisae* NCYC-R397),100 μg; E306 extract of vegetable oils rich in tocopherols, tocopherols, 228 mg; clinoptioliet, 1.64 g, aromatic compounds, 72 mg.

| Gene Symbol | Accession NO. | Nucleotide sequence of primers (5'-3') | Product length |
| --- | --- | --- | --- |
| ACTB | XM_003124280.3 | F: TCTGGCACCACACCTTCT  R: TGATCTGGGTCATCTTCTCAC | 114 |
| TOP2B | NM_001258386.1 | F: AACTGGATGATGCTAATGATGCT  R: TGGAAAAACTCCGTATCTGTCTC | 137 |
| TBP | DQ178129 | F: GATGGACGTTCGGTTTAGG  R: AGCAGCACAGTACGAGCAA | 124 |
| YWHAZ | DQ178130 | F: ATGCAACCAACACATCCTATC  R: GCATTATTAGCGTGCTGTCTT | 137 |
| LPL | NM_214286.1 | F: AACGTCATTGTGGTGGACTGGCT  R: TCCAAGGCTGTATCCCAGGAGGTG | 165 |
| CD36 | NM_001044622 | F: CTGTGGACTCATTGCTGGTGCTG  R: AAAACTGTCTGTAAACTTCCGTGCCT | 179 |
| ACACA | NM_001114269 | F: TGTCCACTCAAGCATACCTCCCA  R: GCTACCATGCCAATCTCATTTCCTCC | 136 |
| FASN | NM_001099930 | F: GCCGAGTACAGCGTCAACAACC  R: TGGTCCTTCTTCATCAGCGGGAT | 172 |
| CPT1B | NM_001007191 | F: AGTCATGGTGGGCGACTAACTATGTG  R: ATCATGGCGTGGACAGCGTTC | 169 |
| PNPLA2 | NM_001098605 | F: CCTGCCTCTCTACGAACTCAAGAGC  R: AGGCTGAACTGGATGCTGGTGT | 132 |
| PRKAA1 | NM_001167633 | F: TCAGGGACTGCTACTCCACAGAGA  R: AAGAGTCAAGTGAGGTTACAGATGAGGT | 136 |
| PRKAA2 | NM_214266 | F: CCAGTGAGTTCTACCTCGCCTCT  R: TGGACATCTTGCTTTAGGGCTGTCT | 140 |
| PPARGC1A | NM_213963 | F: AGACCTGACACAACACGGACAGA  R: TTCAAGAGCAGCAAAAGCATCACAGG | 147 |
| MYH7 | NM_213855 | F: AAGACCCGCTCAACGAGACAGTGG  R: GCCTTGCCTTTGCCCTTCTCAACA | 121 |
| MYH2 | NM_214136 | F: TCATCAGTGCCAACCCGCTG  R: AAGCCAGTTTTCCTGTAGTGCCAAA | 120 |
| MYH4 | NM_001123141 | F: TTGAGGAGTTAAAGAGGCAGCTAGAAGAGG  R: TCGCTGTTGGCCTTGGACATTGC | 166 |
| MYH1 | NM_001104951 | F: TGGAGGCCAGGGTACGTGAA  R: CTTGCGGTCTTCCTCAGTTTGGT | 134 |

Table S2. Primer sequences used to measure gene expression

Table S3. The alpha diversity indices of the microbiota in the feces, caecum and colon contents^a^

| Index | Antibiotic | Control | *p* value |
| --- | --- | --- | --- |
| **Feces** | | | |
| Chao1 | 191.78 ± 8.86 | 187.01 ± 20.05 | 0.831 |
| Shannon | 3.45 ± 0.17 | 3.30 ± 0.19 | 0.541 |
| InvSimpson | 15.36 ± 2.88 | 12.84 ± 2.23 | 0.499 |
| **Caecum content** | | | |
| Chao1 | 226.32± 12.68 | 253.98 ± 10.50 | 0.127 |
| Shannon | 2.88 ± 0.14 | 2.99 ± 0.11 | 0.565 |
| InvSimpson | 8.36 ± 1.24 | 7.86 ± 1.05 | 0.760 |
| **Colon content** | | | |
| Chao1 | 311.65 ± 20.63 | 312.84 ± 18.61 | 0.966 |
| Shannon | 3.87 ± 0.19 | 3.63 ± 0.22 | 0.412 |
| InvSimpson | 23.15 ± 4.49 | 17.56 ± 3.78 | 0.355 |

^a^ Feces samples were collected from piglets before antibiotic treatment, caecum and colon contents were derived from all piglet after 39 days of antibiotic exposure. n=9 for each group.

#
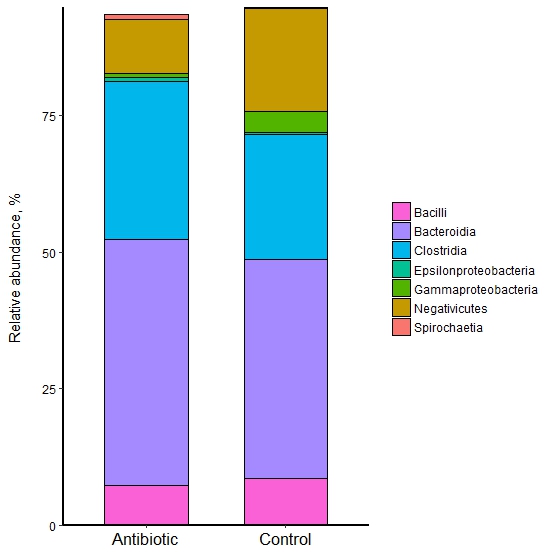
S4 Supplemental figures

Fig. S1. Predominant taxa abundances at class level of the fecal microbiota from piglets before antibiotic treatment. n=9 for each group.


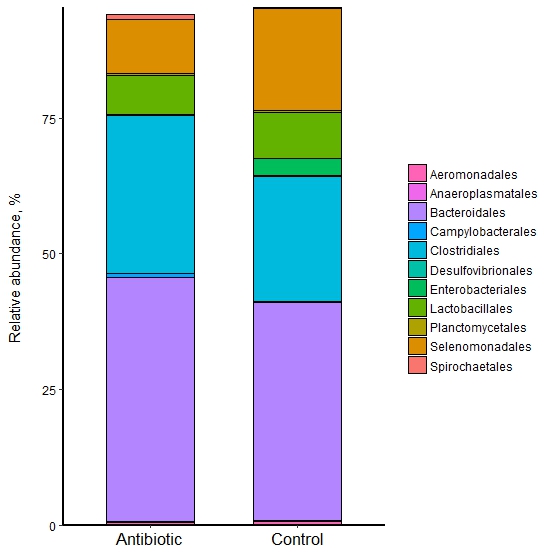
Fig. S2. Predominant taxa abundances at order level of the fecal microbiota from piglets before antibiotic treatment. n=9 for each group.


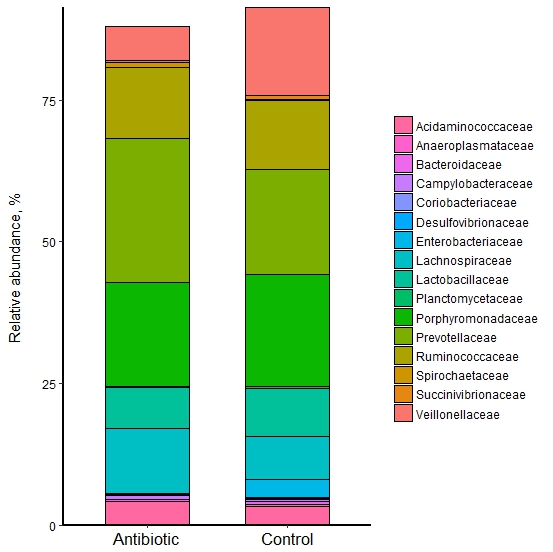
Fig. S3. Predominant taxa abundances at family level of the fecal microbiota from piglets before antibiotic treatment. n=9 for each group.


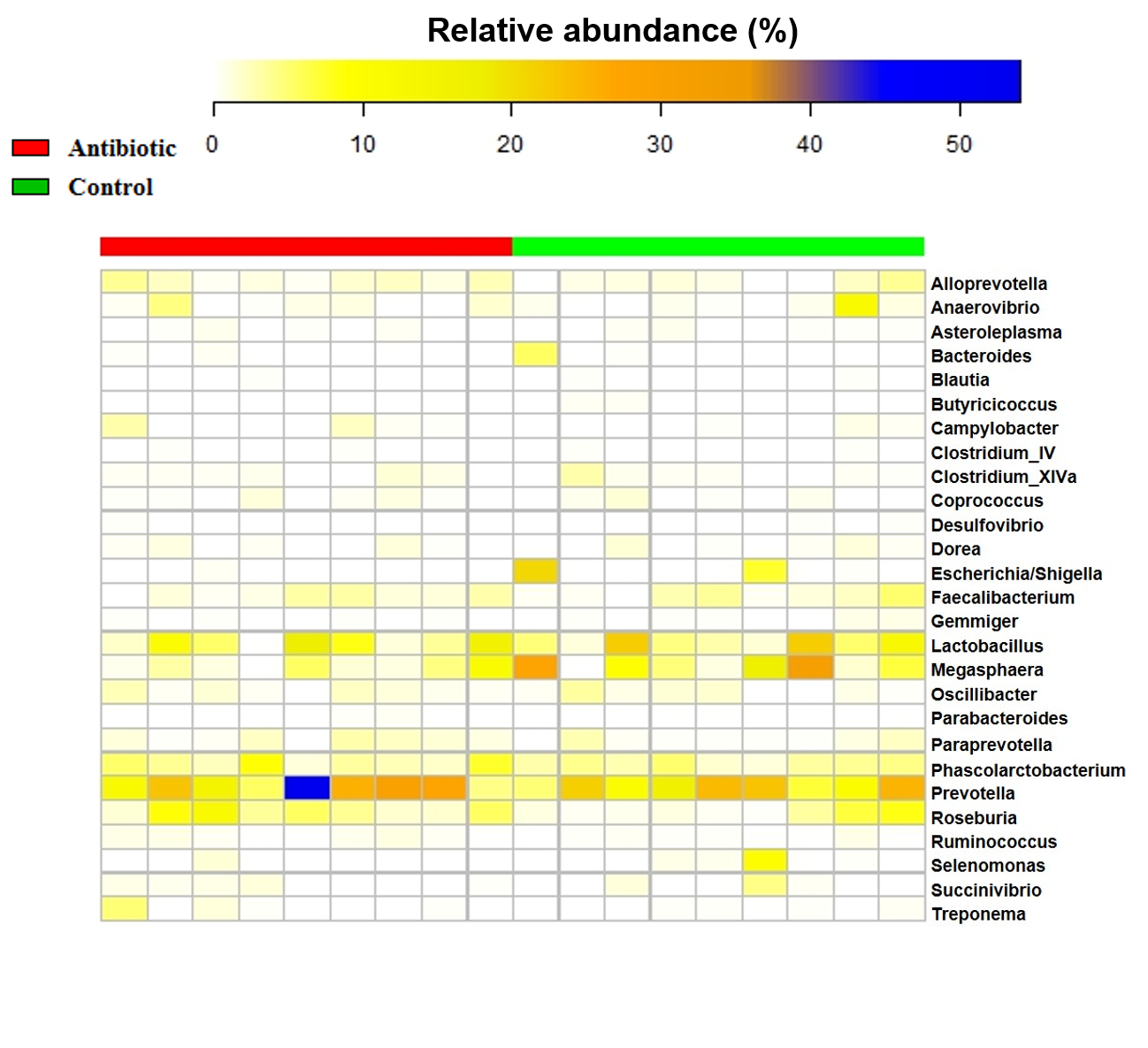
Fig. S4. Heatmap presents the relative abundance of selected genera (average relative abundance more than 0.1% in at least one group) in fecal microbiota of piglets before antibiotic treatment. n=9 for each group.


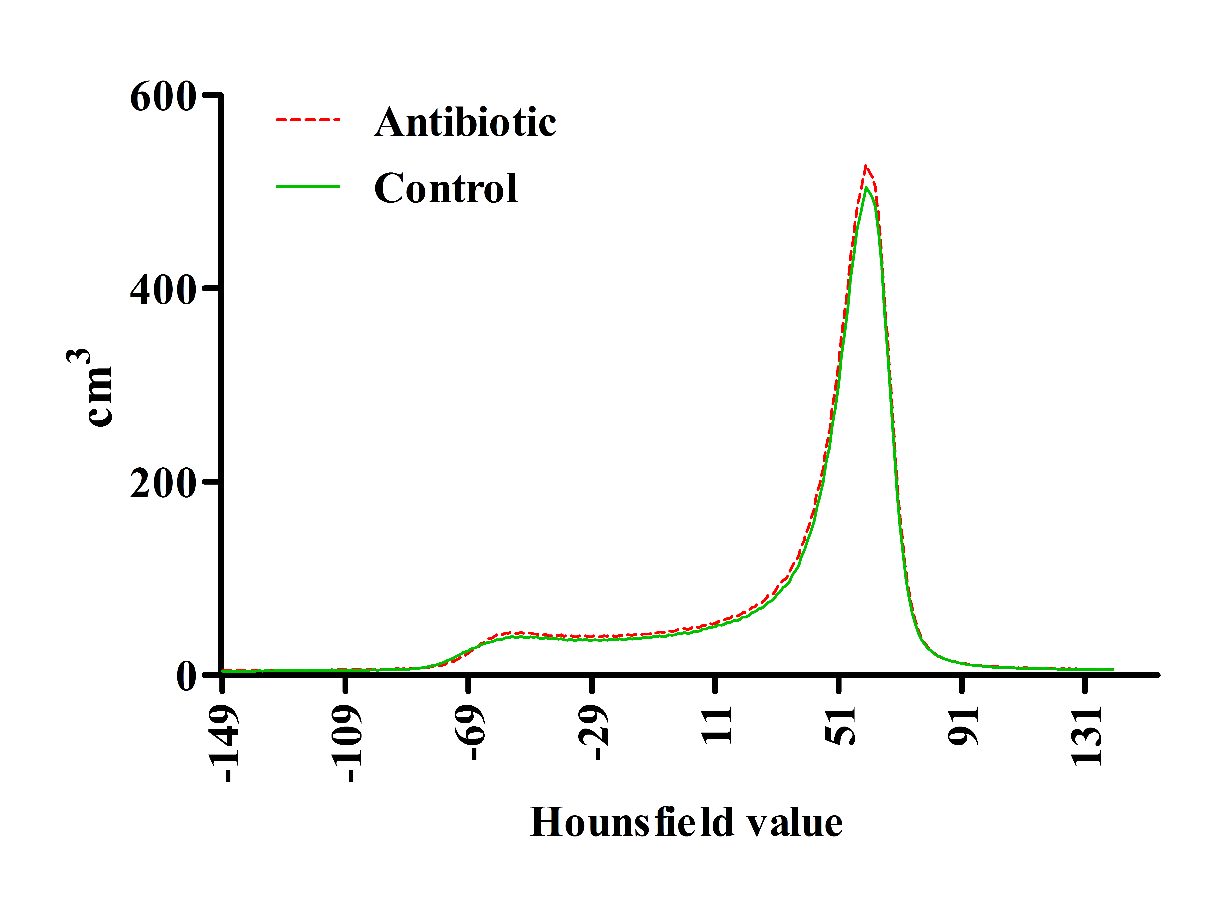
Fig. S5. Distributions of the total volume (cm^3^) per Hounsfield value between the antibiotic and control group. n=9 for each group.


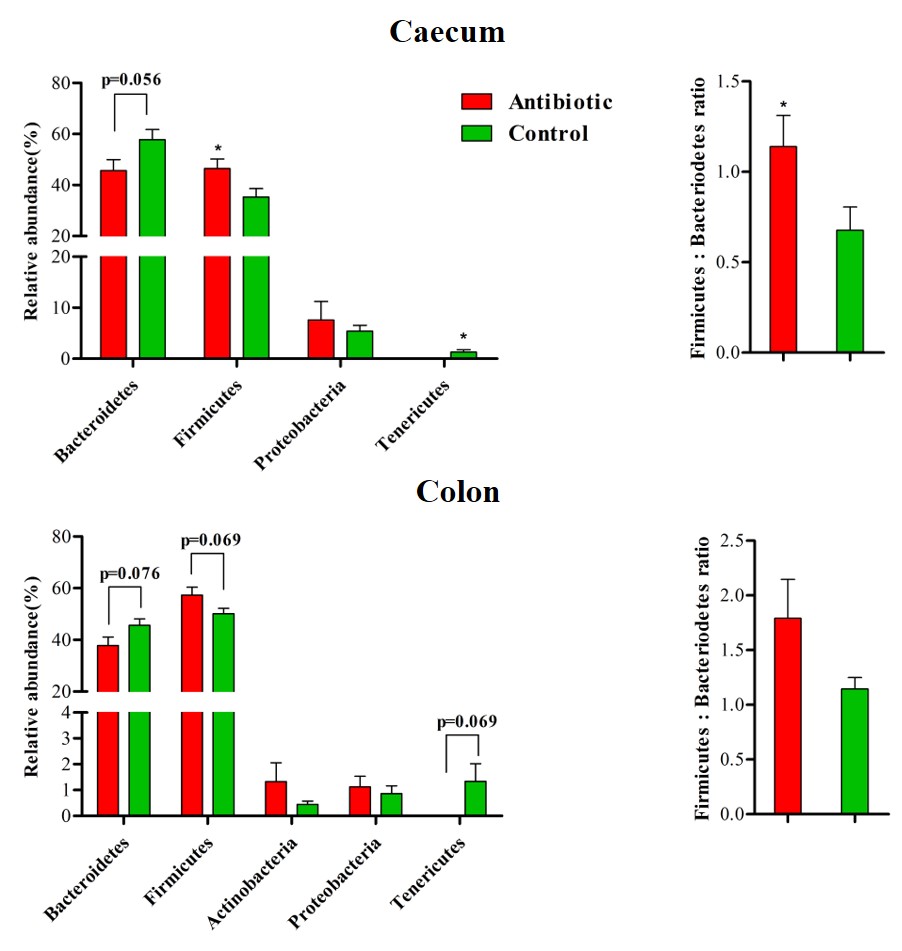


**A**

**B**

Fig. S6. Differences in relative abundances of the predominant phyla and ratio of *Firmicutes* to *Bacteriodetes* in caecal (A) and colonic microbiota (B) between antibiotic-treated piglets and control piglets. *p < 0.05, **p < 0.01. n=9 for each group.

# References

1. i Furnols MF, Gispert M. Comparison of different devices for predicting the lean meat percentage of pig carcasses. Meat Sci. 2009;83:443-446.

2. Font-i-Furnols M, Carabús A, Pomar C, Gispert M. Estimation of carcass composition and cut composition from computed tomography images of live growing pigs of different genotypes. Animal. 2015;9:166-178.

3. Giles L, Eamens G, Arthur P, Barchia I, James K, Taylor R. Differential growth and development of pigs as assessed by X-ray computed tomography. J Anim Sci. 2009;87:1648-1658.

4. Erkens T, Van Poucke M, Vandesompele J, Goossens K, Van Zeveren A, Peelman LJ. Development of a new set of reference genes for normalization of real-time RT-PCR data of porcine backfat and longissimus dorsi muscle, and evaluation with PPARGC1A. BMC Biotechnol. 2006;6:41.

5. Ye J, Coulouris G, Zaretskaya I, Cutcutache I, Rozen S, Madden TL. Primer-BLAST: a tool to design target-specific primers for polymerase chain reaction. BMC Bioinformatics. 2012;13:134.

6. Zuker M. Mfold web server for nucleic acid folding and hybridization prediction. Nucleic Acids Res. 2003;31:3406-3415.

7. Joshi N, Fass J. Sickle: A sliding-window, adaptive, quality-based trimming tool for FastQ files (Version 1.33) [Software]. 2011.

8. Nikolenko SI, Korobeynikov AI, Alekseyev MA. BayesHammer: Bayesian clustering for error correction in single-cell sequencing. BMC Genomics. 2013;14:S7.

9. Masella AP, Bartram AK, Truszkowski JM, Brown DG, Neufeld JD. PANDAseq: paired-end assembler for illumina sequences. BMC Bioinformatics. 2012;13:31.

10. Schloss PD, Westcott SL, Ryabin T, Hall JR, Hartmann M, Hollister EB, et al. Introducing mothur: open-source, platform-independent, community-supported software for describing and comparing microbial communities. Appl Environ Microb. 2009;75:7537-7541.

11. Edgar RC. UPARSE: highly accurate OTU sequences from microbial amplicon reads. Nat Methods. 2013;10:996-998.

12. Edgar RC, Haas BJ, Clemente JC, Quince C, Knight R. UCHIME improves sensitivity and speed of chimera detection. Bioinformatics. 2011;27:2194-2200.

13. Wang Q, Garrity GM, Tiedje JM, Cole JR. Naive Bayesian classifier for rapid assignment of rRNA sequences into the new bacterial taxonomy. Appl Environ Microb. 2007;73:5261-5267.
